# The care‐planning conference: Exploring aspects of person‐centred interactions

**DOI:** 10.1002/nop2.118

**Published:** 2018-01-20

**Authors:** Ingela Jobe, Birgitta Lindberg, Sofi Nordmark, Åsa Engström

**Affiliations:** ^1^ Division of Nursing Department of Health Science Luleå University of Technology Luleå Sweden; ^2^ Health Department Norrbotten Region Luleå Sweden

**Keywords:** aged care, care plan, case study research, documentation, ethics, patient‐centred care, primary care, qualitative approaches

## Abstract

**Aim:**

The aim of this study was to describe the care‐planning conference from the participants' and researchers' perspectives, focusing on exploring aspects of person‐centred interactions.

**Design:**

A single‐instrumental, qualitative case study design was used describing a care‐planning conference taking place in the home of an older woman and her daughter.

**Methods:**

Data collection consisted of observation and digital recording of the care‐planning conference and individual interviews with all the participants before and after the conference. Data were analysed in several phases: first, a narrative description followed by a general description and, thereafter, qualitative content analysis.

**Results:**

The findings revealed that the care‐planning conference conducted had no clear purpose and did not fulfil all parts of the planning process. Three themes emerged related to aspects of person‐centred interactions. The theme “expectations meet reality” showed different expectations, and participants could not really connect during the conference. The theme “navigate without a map” revealed health professionals' lack of knowledge about the care‐planning process. The theme “lose the forest for the trees” described that the conference was conducted only as part of the health professionals' duties. Management and healthcare professionals cannot automatically assume that they are delivering person‐centred care. Healthcare professionals need to be sensitive to the context, use the knowledge and tools available and continuously evaluate and reassess the work carried out.

**What does this paper contribute to the wider global clinical community**

- Persons in need of care planning are often vulnerable in one way or another, and in addition, they are not the ones making demands or requests.
- The primary healthcare clinics and the home‐healthcare services have to make sure that they have a quality assurance system in place with feedback from the services users.

## 1 INTRODUCTION

The ageing population is increasing, and it is becoming more common for older persons with medical conditions to receive care in their own homes (Fjordside & Morville, 2016). Older persons often transition between different levels of health care and between healthcare and community care providers. When transferring from one provider to another, older people are at increased risk of falling through the cracks and experiencing incidents (for example, lack of treatment) and readmission due to poor communication and exchange of information among professionals and between professionals and the patient (Allen, Ottmann, & Roberts, 2013). Organizations and professions have their own legislations, values, responsibilities and budgets, and with this system, there is a risk of fragmentation and lack of continuity of the care provided (Berglund, Hasson, Kjellgren, & Wilhelmson, 2015; Sundström, Petersson, Rämgård, Varland, & Blomqvist, 2017). Collaboration among different professions in the community is complex and multifaceted (Rämgård, Blomqvist, & Petersson, 2015), and studies have identified various barriers and facilitators related to the organization of health care (Jansen, Heijmans, & Rijken, 2015).

Traditionally, spouses, adult children and external family members are the first to assume caregiving responsibilities for older relatives when needs arise. In many economically developed countries, these are the people responsible for coordinating healthcare and social care services with regard to the older person, (Sveriges Kommuner och Landsting, 2016; Toscan, Mairs, Hinton, & Stolee, 2012; Wolff, Spillman, Freedman, & Kasper, 2016) and they are also expected to participate in the decision‐making process (Kildal Bragstad, Kirkevold, & Foss, 2014).

A method used to facilitate collaboration between the health professionals, the patient and the informal caregivers and to integrate the patient's perspective in the decision‐making process and to contribute to person‐centred care is the care‐planning process with the development of a written care plan. Studies have shown that the plan improves self‐management, increases patient centredness and reduces unnecessary care use (Burt et al., 2012; Newbould et al., 2012). However, several studies (Bjerkan, Richter, Grimsmo, Helles, & Brender, 2011; Burt et al., 2012; Jansen et al., 2015; Newbould et al., 2012; Reeves et al., 2014) have shown that the implementation of care planning at primary healthcare clinics is meagre.

This qualitative case study is a part of a larger three‐year project in care planning called “My Plan.” The objectives of the project are to develop improved working methods and to implement products and services to establish coordinated individual planning at discharge from the hospital and in the home/community care and to strengthen the individual's role and make the individual care plan digitally available to individuals through a national e‐health platform where they already have access to their medical records (Region Norrbotten, 2017a).

## 2 BACKGROUND

In Sweden, different authorities and different laws handle primary health care and community care. Primary health care is a part of the county council or region, which supplies municipalities with general practitioners (GP) employed at primary healthcare clinics. The municipalities provide health and community care through home‐healthcare services and employ social workers, homecare aides, Registered Nurses and rehabilitation therapists. According to the legislation (SOU, 2015:20), coordinated individual care planning must take place for patients transferring to another level of care or to the patient's home.

**Figure 1:**
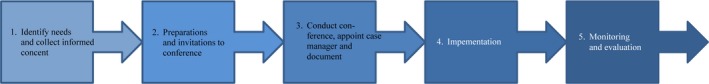
The care‐planning process

The care‐planning process includes different elements; see Figure 1. Before the process can commence, informed consent from the person in focus must be obtained and it is this individual who decides whether or not relatives will participate. The care‐planning conference should result in a coordinated individual plan that clearly states what interventions are needed, who has overall responsibility and how interventions are to be implemented and evaluated (SOU, 2015:20).

The co‐creation of care between patients, their family members, carers and health professionals is also the core component of person‐centred care. Person‐centred care has been shown to advance consensus agreement between care providers and patients with regard to treatment plans, to improve health outcomes and to increase patient satisfaction (Ekman et al., 2011). For care planning to be person centred, the patient's narrative has to be elicited, including identifying the patient's resources, motivations and goals. The patient and the professionals will then agree on a partnership with shared goals and will safeguard this through documentation (Ulin, Olsson, Wolf, & Ekman, 2016).

Research in care planning has focused mainly on inpatient care, and few studies have focused on care planning initiated by the primary healthcare provider and community care. Furthermore, most studies related to care planning have focused on the professionals' perspectives or organizational issues. There is a lack of studies describing the patients' and their relatives' experiences, and there are even fewer studies focusing on person‐centred interactions during the care‐planning conference. The aim of this study was to describe the care‐planning conference from the participants' and researchers' perspectives, focusing on exploring aspects of person‐centred interactions.

## 3 METHOD

The case study provides the means to conduct a holistic, in‐depth and detailed description of a phenomenon using a variety of data collection methods (Merriam, 1998; Stake, 2005; Yin, 2013). For this study, we used a single‐instrumental, qualitative case study design inspired by Stake (2005). According to Stake (2005), a case study is both a process of inquiry and the product of that inquiry. Instrumental case study is used when a particular case is examined to provide insight into an issue, in this case, aspects of person‐centred interactions at the care‐planning conference. The choice of case is made to improve understanding. The qualitative case study aims to understand the particularities of the case from multiple perspectives. The emic issues from the case, the context, meaning and interpretation offer holistic understanding of the case from which we can learn (Abma & Stake, 2014). Using the case study approach, we wanted to know how participants and researchers in a care‐planning conference described aspects of person‐centred interactions.

### 3.1 Context

The care‐planning conference took place in March 2017 in the home of an older woman and her daughter. All the participants were physically present at the conference except for the GP, who participated via video on an iPad. The primary healthcare clinic and municipality participating in the study have been using video communication during care‐planning conferences for the past year, after implementing the use of modern technology in various parts of their daily work through the project RemoAge (Region Norrbotten, 2017b). The municipality is located in the north of Sweden, where the one‐way distance between the primary healthcare clinic and some inhabitants can be more than 80 km.

One week before the care‐planning conference, the primary healthcare clinic had sent a letter to the woman and her relatives inviting them to the meeting. The primary healthcare clinic and the municipality communicate through a communication platform, through which they can send and receive messages and document a coordinated individual care plan together.

The interviews before and after the conference, with the woman and her daughter, took place in their home. A follow‐up interview with the daughter, 1 week after the conference, and the interviews with the health professionals, within 3 weeks after the conference, were all conducted over the phone.

### 3.2 Participants

Participants in the conference were the woman, here called Sara; her daughter; two Registered Nurses (RN1 and RN2) from the home‐healthcare services at the municipality and a general practitioner (GP) from the primary healthcare clinic. A Registered Nurse at the primary healthcare clinic informed the individuals and their relatives of the study verbally and in writing. If they were willing to participate, the author contacted them by phone to give them additional information about the study and to schedule the first interview. The Registered Nurse also informed the health professionals participating in the conference of the study and before the conference commenced, the researchers collected their informed consent.

### 3.3 Data collection and analysis

The care‐planning conference was recorded digitally and the first and last authors observed the care‐planning conference and took notes regarding non‐verbal communication separately. The researchers did not use an observation protocol. Semistructured interviews were conducted with Sara and her daughter before and after the conference and a follow‐up interview over the phone with the daughter took place within a week after the conference. The interview before the care‐planning conference focused on Sara and her daughter's situation, reasons for the conference and their expectations about the conference and the care‐planning process. The interviews after the conference concentrated on their experiences of the care‐planning conference and their subsequent expectations. The two RNs and the GP were interviewed over the phone within 3 weeks after the conference. The semistructured interviews focused on their experiences of the care‐planning conference. The plan established during the care‐planning conference was also going to be a part of the data collection and analysis, but in the studied care‐planning conference, no plan was established.

The first author transcribed verbatim the recorded care‐planning conference and the interviews. The notes taken during the observation were added to the transcript. The data were compiled into a case file. The first, second and last author participated in the analysis process. The data were analysed in several phases, beginning with a narrative description of “Before the care‐planning conference,” followed by a general description of the “Care‐planning conference.” The analysis proceeded with a qualitative content analysis according to Graneheim and Lundman (2004). All the data except for the interviews before the care‐planning conference were part of the analysis. The material was read through several times and the text related to the aim was extracted and divided into meaning units. Meaning units were words, sentences or paragraphs related by content or context. The meaning units were then condensed into codes. The different codes were compared based on similarities and differences and abstracted into categories. Throughout this process, the entire text was continuously read through and taken into consideration to ensure that no aspect or variation in the text was omitted. In all the steps, words in the text, or what the text said, were used for the labelling. The first part of the analysis was done in Swedish, while categories and themes have been labelled in English. The material was read through again several times, and the latent content of the entire text and the categories were formulated into three themes (Table 1).

**Table 1:** Themes and categories

Themes	Categories
Expectations meet reality	Consider the person
	Establish a partnership
	Come to an agreement
	Document the partnership
Navigate without a map	Knowledge of legislation
	Organization of work
Lose the forest for the trees	Management issues
	Power dimensions
	Moral issues

### 3.4 Ethical considerations

Conducting research in the home setting can present ethical challenges that require special considerations. In this qualitative case study, the researchers had the opportunity to interact with and interview Sara and her daughter in their home prior to the care‐planning conference. This made the researchers less threatening during the observation of the conference. However, we are aware that the presence of the researchers may have influenced the findings. The advantages of access to first‐hand data were considered to outweigh the disadvantages. The researchers informed the participants in the care‐planning conference (Sara, her daughter, the RNs and the GP) about the study and collected their informed consent before the study commenced. Descriptions of the participants and the circumstances in the care and life situation have been restricted and pseudonyms have been used to protect all participants' anonymity. The Ethical Regional Board, Umeå, Sweden, granted permission for the study, number (dnr 2016/397‐31).

## 4 FINDINGS

The first part of the findings, titled “Before the care planning conference,” presents the participants and the background that preluded the care‐planning conference based on interviews with all the participants. The second part, titled “Care‐planning conference,” is divided into two sections. The first section, titled “General description,” describes findings based on the data obtained from the observation of the care‐planning conference. The second section, titled “Aspects of person‐centred interactions,” presents findings based on the analysis of all the data in the case file except the interviews conducted before the care‐planning conference.

### 4.1 Before the care‐planning conference

Sara spends her days sitting in her favourite armchair tucked in under a blanket in the living room. She is a small and slim woman who will turn 93 later this year. Her husband passed away in the dawn of the new millennium, she is living with her only child, a daughter and her son‐in‐law. Sara is blind in one eye and has limited sight in the other. She has a hearing impairment and difficulties walking and moving around due to problems with her feet and hypermobile ankles. To move around inside the house, she uses a walker with assistance and outside she uses a wheelchair. Sara takes medication for high blood pressure on a daily basis and painkillers from time to time for general body pain. She is content with her life and believes that she has managed to become this old by eating and sleeping well. She is also grateful to her daughter for letting her stay with them.

Her daughter assists her with personal care and all other practicalities, including contacts and communication with different authorities and services. She also administers her mother's medications and drives her places. Sara's daughter is 59 years old and has a long‐term disease. Her situation has deteriorated, and presently, she is on sick leave and walking with crutches. Her husband is on disability pension after a cerebrovascular accident. All three have enjoyed living together, but as her mother's condition has declined, it has become more difficult. Sara's daughter and her husband have become less independent and their own health has also worsened.

Due to increased difficulties for her daughter and son‐in‐law to provide care for Sara, she has received community care services from homecare aides from the municipality for some months. She receives help both during the day and night, mainly to use the bathroom. Sara is pleased with “the girls” providing the care:

They are very kind and touch my knees so I know they have arrived and it is time for me to fold the blanket and get up. (Sara)

Four months ago, Sara developed pressure wounds on her buttocks and has been going to the primary healthcare clinic regularly to have the wounds dressed. Her daughter has been assisting her and accompanying her there. The preparations and travelling back and forth by taxi (because of her wheelchair), including waiting times, make the journey a full‐day project. It is difficult for Sara to travel and she becomes anxious outside her home. Sometimes she refuses to go, and for some time now, her daughter has not been able to assist her because of her own health situation. The daughter contacted the primary healthcare clinic when she could not assist her mother to travel there, and after that, the primary healthcare clinic initiated the care‐planning conference.

RN1 and RN2 have both been working more than 15 years as primary healthcare nurses, and they work for both the region and municipalities. For the last 1–3 years, they have been working as temporary nurses and this was their third time working for the present municipality. Usually, they stay and work for a couple of weeks each time. The GP has been working as a medical doctor for 5 years. She was employed at the primary healthcare clinic 3 years ago and is presently doing her specialist training in primary healthcare.

### 4.2 Care‐planning conference

#### 4.2.1 General description

**Figure 2:**
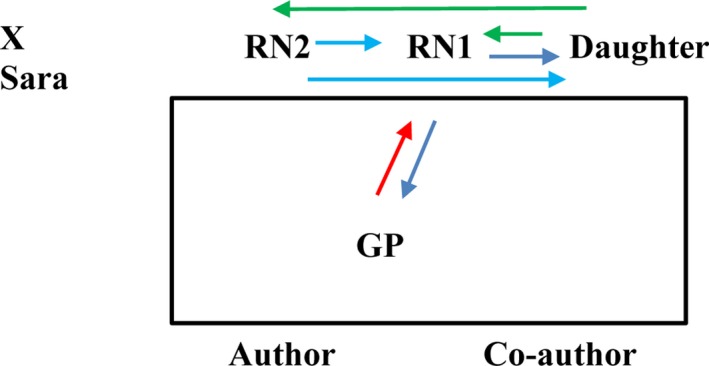
Seating and communication flow

The aim of the care‐planning conference was to transfer the care of the pressure wounds from the primary healthcare clinic to the nurses at the home‐healthcare services for the municipality. The two RNs, together with Sara's daughter, decided at the start of the conference that Sara was not going to participate in the conference due to her hearing impairment. She remained seated in her armchair in the same room but a distance away from the conference table. The conference lasted for approximately 40 min, with the GP participating during the first 10 min. The seating and the communication flow between the participants are illustrated in Figure 2.

There was no appointed chairperson for the conference, but RN1 did most of the talking. The participants used everyday language and the issues discussed appeared to emerge spontaneously; there was no fixed order of speaking. RN1 began the conference by explaining to the GP that she and her colleague did not know the patient. The GP replied that she was in the same situation but could read from the medical records. The GP read from the medical records and informed RN1 of Sara's medical conditions, her current medications, the status of the pressure wounds and her need for care from the nurses at the municipality. When RN1 started asking the daughter questions, the GP interrupted the conversation between RN1 and the daughter to ask if her presence was still needed.

RN1 was the only one communicating with the GP. She informed the daughter about the home‐healthcare services, the care of the pressure wounds and the role of the occupational therapist. She asked the daughter direct questions related to the pressure wounds and present care, the distribution of medicines, interventions from social services and living conditions. RN2 informed the daughter about costs for the services, the need for a nurse to assess the pressure wounds before care could decide and whether nurses or personnel from the social services would be responsible for the dressing. She asked the daughter direct questions related to incontinence care, nutrition and Sara's age. When RN1 returned from a phone conversation, she also informed RN1 of what she had discussed with the daughter. Both nurses took notes during the conference. For the most part, Sara's daughter answered direct questions from the two nurses. She did not ask many questions, but she asked RN2 about the costs of the services and what she should do if her mother deteriorated. She also asked both RN1 and RN2 how her mother would access the plan documented at the conference. She made two statements during the conference. The participants ignored the first one, which was related to difficulties on buying covers and it remained unclear what covers she was referring to. The other statement came at the end of the conference when she explained that she thought home‐healthcare services were included in the social services provided by the municipality.

#### 4.2.2 Aspects of person‐centred interactions

To describe aspects of person‐centred interactions during the care‐planning conference, a qualitative content analysis was performed. The findings of the analysis are presented under the themes: *expectations meet reality*,* navigate without a map and lose the forest for the trees*.

##### Expectations meet reality

The theme “Expectations meet reality” was derived from four categories: consider the person, establish a partnership, make an agreement and document the partnership. The findings revealed that the participants had different expectations and anticipations before the care‐planning conference; they could not really connect during the conference and the outcomes remained unclear after the conference.

*Consider the person*. When the two RNs arrived the house, they greeted Sara sitting in her armchair. After that, Sara was excluded from the conference; her daughter communicated in her place. According to the RNs, children or other relatives speaking for their older parents is a common phenomenon. Sara had a hearing impairment but no cognitive disability. Her daughter wished she could have participated in the meeting:

She trusts me, but maybe she feels that people are talking over her head. For her sake, it would have been better if she could have participated. (Daughter)

Sara wanted to get help with the care of her pressure wounds at home so she would not have to travel to the primary healthcare clinic. She depended on the assistance of her daughter to travel there, and at present, it was not possible for her daughter to assist her. For the daughter, it was also important to get access to the RNs at the home‐healthcare services in the event that Sara's condition deteriorated. However, the RNs wanted to focus on the present issues and not discuss other concerns:

If things will change, I just need to inform you or? (Daughter)

What did you say? (RN2)

If her situation deteriorates and she will need more assistance. (Daughter)

Presently, we have this situation. (RN2)

You have to excuse us, but the phones are like this. (RN1 interrupts on returning from a phone conversation and after that, the topic changes.)

*Establish a partnership*. None of the healthcare professionals participating in the conference had met Sara or her daughter before. They based their knowledge on second‐hand information, information from the medical record and by asking her daughter questions. The daughter commented on this and on the constant change in personnel:

I was surprised that it was not nurses from here or the GP we met… so we hope things will go well (Daughter, laughing)

Both the primary healthcare clinic and the municipality used temporary staff on a regular basis due to vacant positions for GPs and RNs:

It would be better and more fun to only participate in the care‐planning conferences with your own patients, but that is not possible due to the lack of GPs. (GP)

The daughter also lacked knowledge about the home‐healthcare services, their role and responsibilities. She thought the home‐healthcare services were included in the social care her mother received.

The daughter thought not only the other participants listened to her but she also acknowledged that she did not have a lot to say and that although she participated in the conference, she was not really part of it:

I think they listened, yes, I was sitting there…. (Daughter)

*Come to an agreement*. The RNs used most of the conference time to collect information about Sara from the GP and the daughter. From the observers' perspectives, it was not obvious if or when decisions were made during the conference. After the conference, the daughter had many questions and she was not clear about the outcomes. She had, for example, not understood if Sara's needs now belonged to the home‐healthcare services or if she only had access to them for a period until the pressure wounds healed. She did not know if it was her or the RNs who should contact the occupational therapist and make an appointment. She had not received any contact details for the home‐healthcare services, so she had no way to contact them and her questions had to wait until they visited again.

*Document the partnership*. Prior to the care‐planning conference, Sara had received a letter from the primary healthcare clinic. Apart from practical information about date, time and place, it said that a copy of the plan developed during the conference would be sent to her afterwards. However, the RNs at the home‐healthcare services did not have the same understanding regarding the documentation made of the care‐planning conference as stated in the letter sent to Sara. The RNs and the GP documented only in their respective journal systems and none of them documented a plan with agreements about the outcomes and who had the overall responsibilities that they could share with Sara. Therefore, Sara never received any plan:

How will she get access to the plan? (Daughter)

Now let us see if we can answer that… hmm… (RN2)

I do not think it is something common that the patient or relative will be sent a copy, but of course if you request it… (RN1)

##### Navigate without a map

The theme “Navigate without a map” was derived from two categories: knowledge of legislation and organization of work. The findings revealed that none of the health professionals had knowledge about the care‐planning process and even if the two principals had established a local organization for carrying out the care‐planning conference, it was evident that the individual health professionals participating were not aware of all parts of the organization and were not satisfied with the collaboration.

*Knowledge of legislation*. None of the health professionals had knowledge about the legislation governing care planning or the different parts of the planning process. During the care‐planning conference, there was no evidence of preparations, no case manager appointed and no plan documented. Through the interviews, it became obvious that they also had no knowledge regarding the monitoring and evaluation of the plan. The health professionals used the care‐planning conference to transfer care of Sara's pressure wounds between them, but they did not consider her need for a documented plan with clear outcomes and contact details:

I only do what I think I should do, I have never evaluated if I do right or wrong and no one has told me how I should do care planning. I have just learnt by practicing. (GP)

The RNs said that they were not aware of any structure for the care‐planning process and that they had never received any education or training in care planning. They did not know if their present workplace had guidelines for conducting a care‐planning conference or for how to collaborate with other actors around the same patient. Nonetheless, all of the health professionals felt confident in their roles during care planning.

*Organization of work*. The health professionals belonged to different principals, the region and the municipality. They used the care‐planning conference to transfer patients from the region to the municipality. However, the criteria for transfer of patients were unclear and the different principals interpreted the criteria differently. The RNs said that, many times, the primary healthcare clinic wanted to transfer patients who did not meet the criteria to become a home‐healthcare patient. With two different principals, organizational issues and economic factors became more important than what was best for each patient:

Sara did not fulfil the criteria for home‐healthcare service. I visited her the day after the conference to assess the pressure wounds and the outcome of the conference was not correct. She did not qualify to become a home‐healthcare patient, not if you follow the threshold principle. (RN2)

Both the RNs and the GP talked a lot about the difficulties of working together and collaborating. They thought that it would have been better for the patients if there had been only one principal or if they at least had the same medical record system:

With two different principals, it is not safe for the patient and the access to the GP is very complicated. It would be easier if we had the same journal system; at the moment, we do not have access to a current medication list and we are travelling with post‐it notes back and forth between us and the primary healthcare clinic. (RN1)

During the care‐planning conference, they used video communication to facilitate the participation of different participants, to save time and to create opportunities for more care‐planning conferences to take place. The daughter said that they talked a lot with the iPad. The RNs did not support the use of iPads. They thought that it prevented many older persons from participating in the conference and those who did not hear well or understand what the GP said were afraid to ask questions. The GP acknowledged that the care planning was for the patient and not for the health professionals and that it was important to find a way to conduct the conference according to what would be best for each individual patient:

It is not good if the person cannot participate or will not speak due to their old age and does not understand that it is the GP on the screen of the iPad. If that is the case, I do not think we should use the iPad. (GP)

##### Lose the forest for the trees

The theme “Lose the forest for the trees” was derived from three categories: management issues, power dimensions and moral issues. The findings revealed that the health professionals conducted the care‐planning conference as part of their duties, but the focus of the care planning, that is, Sara, was nowhere to be found.

*Management issues*. The two principals had established an organization to conduct the care‐planning conference and to use video communication; however, not all parts of the process were included in the care planning. It was also unclear who was responsible for connecting the dots and making sure that all the parts would take place. From a person‐centred perspective, the health professionals made an attempt to get to know Sara, but they failed to establish a partnership and made no documentation.

The temporary RNs at the municipality did not have access to the communication platform used to communicate and share information between the region and the municipality regarding care planning; therefore, they encountered problems carrying out their part of the work.

The conference did not follow an agenda and it was not possible to detect any structure of the meeting with a clear beginning or end. The conversation between the participants was interrupted several times when the nurses' mobile phones rang and when the RNs or GP interrupted each other. Consequently, some topics discussed during the conference were never concluded and some of the information provided was not followed up. The RNs, for example, never asked Sara's daughter if she understood everything or if she wanted to ask any questions.

*Power dimensions*. The care‐planning conference was not an arena where individuals participated on equal terms. Sara was depending on her daughter and the health professionals and her daughter was speaking for her during interactions with the health professionals and depending on the professionals. Both were happy to get help for Sara in the home, so they did not need to travel to the primary healthcare clinic. Sara did not participate at all, either in the conference or in the decision‐making process and her daughter played a minor role. Her daughter was more active at the beginning of the conference, but after the other participants ignored a statement she made, she mainly answered direct questions and did not take initiative. On several occasions, the RNs spoke to the daughter but did not seem to expect any response. For example, one of the RNs explained that they needed to come back the following day to assess Sara's pressure wounds:

You are home during the day, so it does not matter what time we come.

We usually never say a time; it is difficult to say if we will come in the morning or in the afternoon because things keep on changing and we have to make adjustments the whole time. (RN2)

The RNs felt pressure and demands from Sara's daughter and from the GP. On the one hand, they thought that it was difficult to say no to the GP's demand that Sara be made a home‐healthcare patient, even if they knew that they had right to do so according to the threshold principle. On the other hand, it was equally difficult for them to say no to Sara and her daughter and deny them the care they needed and wanted:

It is difficult as a nurse to say no or to decide against the GP. (RN1)

We have the right to question the GP's decision, but how easy is it to say to the patient and the family that, no, you will not become a home‐healthcare patient. (RN2)

*Moral issues*. By inviting individuals to a care‐planning conference, the healthcare professionals are creating expectations. Sara and her daughter said that they did not have any expectations of the conference, but the daughter asked about the plan at the end of the meeting. According to the letter they received before the conference, a coordinated individual plan was going to be developed during the conference.

One of the RNs acknowledged that they could have attempted to include Sara's participation in the conference by sitting next to her and talking with her, but for one reason or another, they choose not to do that:

Of course, we could have been sitting next to her where she was sitting in her armchair and talked with her; that we could have done, eh, but now we did not do that, hmm… (RN1)

According to the GP, care planning was something she had to do; but personally, she did not feel a strong commitment to it:

It is something we have to do. I do not really know what to say; when you ask these questions, I feel I do not have a personal commitment to this. (GP)

## 5 DISCUSSION

As observers of the care‐planning conference, we asked ourselves several questions. For whom was the conference conducted? What was the purpose? Should this kind of care‐planning conferences take place at all? The findings show that not all care‐planning conferences conducted have a clear purpose or follow a structured agenda to ensure that all the different parts are included. They also confirm the complexity when different actors are collaborating, as revealed in other studies (Jansen et al., 2015; Rämgård et al., 2015). In Sweden, the law (SOU, 2015:20) regulates care‐planning collaboration. However, the law leaves room for interpretation and it is up to each care provider to create the conditions required for collaboration. This means that the care‐planning process and how the care‐planning conference is conducted vary from municipality to municipality. In addition, in many municipalities, the management and professionals are not knowledgeable about the law. If the actors are not able to collaborate fully and not all parts of the care‐planning process are achieved, this will affect the patient. In this case, it affected Sara, who never received a coordinated individual care plan.

The care‐planning process and delivering a person‐centred approach to care follow the same principles (Ekman et al., 2011; Moore et al., 2016; SOU, 2015:20) and both struggle to achieve their purpose. A common difficulty is the involvement of the patient and/or informal caregiver in the decision‐making process. Sara herself did not participate in the conference and her daughter was involved in the decision‐making process only to a certain degree. A study by Bragstad et al. (2014) showed that informal caregivers take on the role of active participants on behalf of their older relatives when meeting with healthcare services and struggle to establish dialogues and gain influence over decisions.

The health professionals dominated and controlled the conference. Sara's perspective of herself and her life situation was left out and the participants communicated about her but not with her. This concurs with findings by Efraimsson, Rasmussen, Gilje, and Sandman (2003) regarding professional carers dominating the conversation and the patient experiencing a feeling of being treated as an object. Berglund, Dunr, Blomberg, and Kjellgren (2012) found that the location for the care‐planning conference, whether it took place in the patient's home or in the hospital, had no impact on the patient's ability to influence the decision‐making process. The organizational processes seem to be built into the care‐planning system. In the person‐centred approach to care, each person is unique, but a shift of approaches cannot happen by itself. Barriers to person‐centred care include traditional practices and structures and professionals' attitudes. According to Moore et al. (2016), studies propose that the organizational elements and paternalism in Swedish healthcare prevent patients' participation despite legal requirements. This study confirms that the healthcare professionals need help and support to make the shift to person‐centred care and to be able to involve the individual in the decision‐making process during care planning.

To participate in care planning, person‐centred care and decision‐making create demands on the individual person and the informal caregivers. This can be challenging for older persons, like Sara and her daughter, who have learnt to interact with their healthcare professionals in a paternalistic way. Participation and influence relate to issues associated with power and dependency. The professionals have to invite, in this case, Sara and her daughter to participate on equal terms and give them power. According to Gibson (1991), nurses should use their power as a tool for empowerment in the context of an equal partnership with patients. Nurses need to surrender the need for control and accept that patients will make decisions that are different from those decided for them. In the study by Berglund et al. (2012), the professionals initiated most of the conversation during care planning and concerns brought by the older people that fell outside the predefined agenda were not completed; the professionals controlled the discussion and proposed solutions, which align with the findings in this study.

Involving Sara and her daughter in decision‐making is an ethical dilemma and places great demands on the professionals. Research describes how older persons experience less influence when their dependency increases and that they have to organize their life around their carer's schedule. There is a gap between the carer's ethical ideal and actual actions and carers experience a conflict of interest in balancing the older person's rights against external demands (Fjordside & Morville, 2016). According to Buber (1997), every human being wants to be confirmed by others. A mutual relationship is realized the moment that we succeed in making the other present as a person to us and understand his or her experience of the current situation. Sara is depending on her daughter as the one person who knows her best; together, they are a team with a shared narrative. The health professionals failed to confirm them during the encounter. Naldemirci et al. (2016) call for a broader definition of narrative so that it can be seen as jointly constructed by the patient, the family members, the caregivers and symbolic and material aids. The focus should be on the interaction that connects different actors rather than on the individual person. The health professionals did not recognize Sara as a capable and resourceful person. To practice person‐centred care, they should have seen Sara, the individual and created the right conditions for her to be able to participate in the care‐planning conference. According to Jonasson, Liss, Westerlind, and Berterö (2010), ethical values include being receiving, inviting and showing professionalism. When the health professionals' behaviours and actions are in harmony with these ethical values, the patient will feel valued and be allowed to interact on equal terms. However, Kitson, Marshall, Bassett, and Zeitz (2013) question whether personal qualities including good manners and respect are the foundation of person‐centred care and whether health professionals who are not able to practice this or to recognize the importance of relationships are able to deliver person‐centred care.

The failure of the health professionals to consider Sara and to establish a partnership with her and her daughter affected the conference and its outcomes, for example, the daughter's confusion and uncertainty about what had been decided. To be able to establish a partnership on equal terms, Sara and her daughter needed more knowledge about the roles and responsibilities of the different actors and about the care‐planning process, in addition to tools that would have assisted them in preparing for the conference and enabled them to participate on equal terms. Apart from providing information, the health professionals should also find out to what degree the individuals have the capacity to obtain, process and understand the information and services needed to make appropriate decisions. By empowering people, their health literacy will improve (World Health Organization, 2013). According to Sak, Rothenfluh, and Schulz (2017), people who report a higher level of psychological empowerment and health literacy preferred and assumed greater participation in medical decisions.

There are different tools available to assist the participation of individuals and the carers in care planning. In a study by Sundström et al. (2017), family members expressed that an agenda for the care‐planning conference could have provided a tool for them to discuss ideas and preferences before the meeting as most of them came unprepared. One tool that is used to identify goals of homecare recipients is TARGET (Towards Achieving Realistic Goal in Elders Tool), which has shown a greater proportion of goal identification, higher rates of goal attainment and a more diverse range of goals than non‐structured methods of goal setting (Parsons & Parsons, 2012). Another way to assist the older person and to support his or her relatives more active participation in care planning is the appointment of a case manager or coordinator. This has shown to not only increase patient satisfaction and reduce costs but also facilitate coordination and communication and improve transitions between providers (Hudon, Chouinard, Diadiou, Lambert, & Bouliane, 2015). The use of case managers or coordinators has improved knowledge of who to contact about care and services and also provided support for the informal carers (Berglund et al., 2013).

### 5.1 Methodological considerations

Stake (2005) has a constructivist approach to case study that fitted our intention with the research and philosophical orientation. It provided guidance without creating restrictions. The case, in our study the care‐planning conference, was instrumental and facilitated our understanding and provided insight into aspects of person‐centred interactions. We can also learn from it. The use of multiple data sources provided rich data. However, using an observation protocol with different dimensions, we could probably have collected more information during the observations. A case study is suitable for illuminating the particularities and complexity of human phenomena. According to Abma and Stake (2014) in capturing the essence and uniqueness of the case, it will reveal something that is universal.

## 6 CONCLUSION

The qualitative case study conducted confirms the complexity of collaboration between different actors. It also highlights the importance of being attentive to our own practice. Management and healthcare professionals cannot assume that they are delivering person‐centred care automatically. There is “no one size fits all” solution. Professionals need to be sensitive to the context, use the knowledge and tools available and continuously evaluate and reassess the work carried out. There are numerous resources invested in care planning and if the process is not beneficial to the participants, we have to ask ourselves why we carry it out and how we can make it function for all participants.

## 7 RELEVANCE TO CLINICAL PRACTICE

Every human being wants to be affirmed by others by being seen, heard and taken seriously. Healthcare professionals must be professional in all encounters with patients and their informal caregivers. They need to spend enough time and have adequate knowledge to prepare for an effective meeting and to create a trustful and enabling environment with the right conditions for the individual person to be capable and able to participate as a partner in the care‐planning process. The management has to promote these goals and provide time for the healthcare professionals to carry out this important work. The management also has to support the healthcare professionals on different levels to make the shift to person‐centred care. The professionals need to continuously discuss and reflect on ethical issues and aspects of person‐centred care to be able to adjust different contexts and individuals so that care‐planning conferences like the one in this qualitative case study will not continue to take place.

## CONFLICT OF INTEREST

We declare there is no conflict of interest.

## AUTHOR CONTRIBUTIONS

IJ, ÅE, BL and SN were involved in study design; IJ and ÅE collected the data; IJ, ÅE, BL and SN performed the data analysis; IJ, ÅE, BL and SN were involved in manuscript preparation.

## References

[nop2118-bib-0001] Abma, T. A. , & Stake, R. E. (2014). Science of the particular. Qualitative Health Research, 24(8), 1150–1161. https://doi.org/10.1177/1049732314543196 25028158 10.1177/1049732314543196

[nop2118-bib-0002] Allen, J. , Ottmann, G. , & Roberts, G. (2013). Multi‐professional communication for older people in transitional care: A review of the literature. International Journal of Older People Nursing, 8(4), 253–269. https://doi.org/10.1111/j.1748-3743.2012.00314.x 22309308 10.1111/j.1748-3743.2012.00314.x

[nop2118-bib-0003] Berglund, H. , Dunr, A. , Blomberg, S. , & Kjellgren, K. (2012). Care planning at home: A way to increase the influence of older people. International Journal of Integrated Care, 12(5), e134 https://doi.org/10.5334/ijic.817 23593048 10.5334/ijic.817 PMC3601533

[nop2118-bib-0004] Berglund, H. , Hasson, H. , Kjellgren, K. , & Wilhelmson, K. (2015). Effects of a continuum of care intervention on frail older persons' life satisfaction: A randomized controlled study. Journal of Clinical Nursing, 24(7–8), 1079–1090. https://doi.org/10.1111/jocn.12699 25293644 10.1111/jocn.12699

[nop2118-bib-0005] Berglund, H. , Wilhelmson, K. , Blomberg, S. , Dunér, A. , Kjellgren, K. , & Hasson, H. (2013). Older people's views of quality of care: A randomised controlled study of continuum of care. Journal of Clinical Nursing, 22(19–20), 2934–2944. https://doi.org/10.1111/jocn.12276 23808647 10.1111/jocn.12276

[nop2118-bib-0006] Bjerkan, J. , Richter, M. , Grimsmo, A. , Helles, R. , & Brender, J. (2011). Integrated care in norway: The state of affairs years after regulation by law. International Journal of Integrated Care [Serial Online], 11 https://doi.org/10.5334/ijic.530 10.5334/ijic.530 PMC3107091 21637705

[nop2118-bib-0007] Bragstad, L. K. , Kirkevold, M. , & Foss, C. (2014). The indispensable intermediaries: A qualitative study of informal caregivers' struggle to achieve influence at and after hospital discharge. BMC Health Services Research, 14(1), 331 https://doi.org/10.1186/1472-6963-14-331 25078610 10.1186/1472-6963-14-331 PMC4119054

[nop2118-bib-0008] Buber, M. (1997). Distans och relation (P. Sällström, Trans. Distanz und beziehung, 1951 (Distance and relation)). Dualis Förlag AB, Ludvika, Sweden.

[nop2118-bib-0009] Burt, J. , Roland, M. , Paddison, C. , Reeves, D. , Campbell, J. , Abel, G. , & Bower, P. (2012). Prevalence and benefits of care plans and care planning for people with long‐term conditions in england. Journal of Health Services Research & Policy, 17(1 suppl), 64–71. https://doi.org/10.1258/jhsrp.2011.010172 22315479 10.1258/jhsrp.2011.010172

[nop2118-bib-0010] Efraimsson, E. , Rasmussen, B. H. , Gilje, F. , & Sandman, P. (2003). Expressions of power and powerlessness in discharge planning: A case study of an older woman on her way home. Journal of Clinical Nursing, 12(5), 707–716. https://doi.org/10.1046/j.1365-2702.2003.00718.x 12919217 10.1046/j.1365-2702.2003.00718.x

[nop2118-bib-0011] Ekman, I. , Swedberg, K. , Taft, C. , Lindseth, A. , Norberg, A. , Brink, E. , … Kjellgren, K. (2011). Person‐centered care—Ready for prime time. European Journal of Cardiovascular Nursing, 10(4), 248–251. https://doi.org/10.1016/j.ejcnurse.2011.06.008 21764386 10.1016/j.ejcnurse.2011.06.008

[nop2118-bib-0012] Fjordside, S. , & Morville, A. (2016). Factors influencing older people′ s experiences of participation in autonomous decisions concerning their daily care in their own homes: A review of the literature. International Journal of Older People Nursing, 11(4), 284–297. https://doi.org/10.1111/opn.12116 27019374 10.1111/opn.12116

[nop2118-bib-0013] Gibson, C. H. (1991). A concept analysis of empowerment. Journal of Advanced Nursing, 16(3), 354–361. https://doi.org/10.1111/j.1365-2648.1991.tb01660.x 2037742 10.1111/j.1365-2648.1991.tb01660.x

[nop2118-bib-0014] Graneheim, U. H. , & Lundman, B. (2004). Qualitative content analysis in nursing research: Concepts, procedures and measures to achieve trustworthiness. Nurse Education Today, 24(2), 105–112. https://doi.org/10.1016/j.nedt.2003.10.001 14769454 10.1016/j.nedt.2003.10.001

[nop2118-bib-0015] Hudon, C. , Chouinard, M. , Diadiou, F. , Lambert, M. , & Bouliane, D. (2015). Case management in primary care for frequent users of health care services with chronic diseases: A qualitative study of patient and family experience. The Annals of Family Medicine, 13(6), 523–528. https://doi.org/10.1370/afm.1867 26553891 10.1370/afm.1867 PMC4639377

[nop2118-bib-0016] Jansen, D. L. , Heijmans, M. , & Rijken, M. (2015). Individual care plans for chronically ill patients within primary care in the netherlands: Dissemination and associations with patient characteristics and patient‐perceived quality of care. Scandinavian Journal of Primary Health Care, 33(2), 100–106. https://doi.org/10.3109/02813432.2015.1030167 25961964 10.3109/02813432.2015.1030167 PMC4834496

[nop2118-bib-0017] Jonasson, L. , Liss, P. , Westerlind, B. , & Berterö, C. (2010). Ethical values in caring encounters on a geriatric ward from the next of kin's perspective: An interview study. International Journal of Nursing Practice, 16(1), 20–26. https://doi.org/10.1111/j.1440-172X.2009.01805.x 20158544 10.1111/j.1440-172X.2009.01805.x

[nop2118-bib-0018] Kitson, A. , Marshall, A. , Bassett, K. , & Zeitz, K. (2013). What are the core elements of patient‐centred care? A narrative review and synthesis of the literature from health policy, medicine and nursing. Journal of Advanced Nursing, 69(1), 4–15. https://doi.org/10.1111/j.1365-2648.2012.06064.x 22709336 10.1111/j.1365-2648.2012.06064.x

[nop2118-bib-0019] Merriam, S. B. (1998). Qualitative research and case study applications in education. (Rev. and expanded ed.). San Francisco: Jossey‐Bass.

[nop2118-bib-0020] Moore, L. , Britten, N. , Lydahl, D. , Naldemirci, Ö. , Elam, M. , & Wolf, A. (2016). Barriers and facilitators to the implementation of person‐centred care in different healthcare contexts. Scandinavian Journal of Caring Sciences, 31, 662–673. Epub ahead of print https://doi.org/10.1111/scs.12376 27859459 10.1111/scs.12376 PMC5724704

[nop2118-bib-0021] Naldemirci, O. , Lydahl, D. , Britten, N. , Elam, M. , Moore, L. , & Wolf, A. (2016). Tenacious assumptions of person‐centred care? exploring tensions and variations in practice. Health: An Interdisciplinary Journal for the Social Study of Health, Illness and Medicine, 22, 54–71. https://doi.org/10.1177/1363459316677627 10.1177/1363459316677627 27879342

[nop2118-bib-0022] Newbould, J. , Burt, J. , Bower, P. , Blakeman, T. , Kennedy, A. , Rogers, A. , & Roland, M. (2012). Experiences of care planning in england: Interviews with patients with long term conditions. BMC Family Practice, 13(1), 71 https://doi.org/10.1186/1471-2296-13-71 22831570 10.1186/1471-2296-13-71 PMC3436749

[nop2118-bib-0023] Parsons, J. G. , & Parsons, M. J. (2012). The effect of a designated tool on person‐centred goal identification and service planning among older people receiving homecare in new zealand. Health & Social Care in the Community, 20(6), 653–662. https://doi.org/10.1111/j.1365-2524.2012.01081.x 22812380 10.1111/j.1365-2524.2012.01081.x

[nop2118-bib-0024] Rämgård, M. , Blomqvist, K. , & Petersson, P. (2015). Developing health and social care planning in collaboration. Journal of Interprofessional Care, 29(4), 354–358. https://doi.org/10.3109/13561820.2014.1003635 25633427 10.3109/13561820.2014.1003635

[nop2118-bib-0025] Reeves, D. , Hann, M. , Rick, J. , Rowe, K. , Small, N. , Burt, J. , & Richardson, G. (2014). Care plans and care planning in the management of long‐term conditions in the UK: A controlled prospective cohort study. British Journal of General Practice, 64(626), e575 https://doi.org/10.3399/bjgp14x681385 10.3399/bjgp14X681385 PMC4141614 25179071

[nop2118-bib-0026] Region Norrbotten . (2017a). Min plan. My plan. Available from: http://www.norrbotten.se/sv/Utveckling-och-tillvaxt/Utvecklinginom-halso-och-sjukvard/Utvecklingssatsningar/Min-plan/ [last accessed 30‐06‐2017].

[nop2118-bib-0027] Region Norrbotten . (2017b). RemoAge. Available from http://www.norrbotten.se/sv/Utveckling-och-tillvaxt/Utveckling-inom-halso-ochsjukvard/Utvecklingssatsningar/RemoAge/ [last accessed 30‐06‐2017].

[nop2118-bib-0028] Sak, G. , Rothenfluh, F. , & Schulz, P. J. (2017). Assessing the predictive power of psychological empowerment and health literacy for older patients' participation in health care: A cross‐sectional population‐based study. BMC Geriatrics, 17(1), 91 https://doi.org/10.1186/s12877-017-0448-x 28219334 10.1186/s12877-017-0448-x PMC5319152

[nop2118-bib-0029] SOU . (2015:20). Trygg och effektiv utskrivning från sluten vård. Betänkande av utredning om betalansvarslagen. [Safe and efficient discharge from inpatient care. Report of the investigation of the Pay‐ment Responsibility Act]. Stockholm: Ministry of Health and Social Affairs.

[nop2118-bib-0030] Stake, R. E. (2005). Qualitative case studies In Norman K. D. & Yvonna S. L. (Eds.), The Sage handbook of qualitative research (pp. 443–446). Thousand Oaks, CA: Sage, cop.

[nop2118-bib-0031] Sundström, M. , Petersson, P. , Rämgård, M. , Varland, L. , & Blomqvist, K. (2017). Health and social care planning in collaboration in older persons' homes: The perspectives of older persons, family members and professionals. Scandinavian Journal of Caring Sciences, 27, 246 https://doi.org/10.1111/scs.12440 10.1111/scs.12440 28543670

[nop2118-bib-0032] Sveriges Kommuner och Landsting . (2016) Samordnad individuell plan för äldre (SIP). När det behövs samordning. [Coordinated individual plan for elderly persons (CIP). When coordination is needed]. Print: LTAB

[nop2118-bib-0033] Toscan, J. , Mairs, K. , Hinton, S. , & Stolee, P. , & InfoRehab Research Team . (2012). Integrated transitional care: Patient, informal caregiver and health care provider perspectives on care transitions for older persons with hip fracture. International Journal of Integrated Care, 12(13), 1–14. https://doi.org/10.5334/ijic.797 10.5334/ijic.797 PMC3429139 22977426

[nop2118-bib-0034] Ulin, K. , Olsson, L. , Wolf, A. , & Ekman, I. (2016). Person‐centred care–An approach that improves the discharge process. European Journal of Cardiovascular Nursing, 15(3), e26 https://doi.org/10.1177/1474515115569945 10.1177/1474515115569945 25648848

[nop2118-bib-0035] Wolff, J. L. , Spillman, B. C. , Freedman, V. A. , & Kasper, J. D. (2016). A national profile of family and unpaid caregivers who assist older adults with health care activities. JAMA Internal Medicine, 176(3), 372–379. https://doi.org/10.1001/jamainternmed.2015.7664 26882031 10.1001/jamainternmed.2015.7664 PMC4802361

[nop2118-bib-0036] World Health Organization . (2013). Health Literacy. The Solid Facts. Copenhagen, Denmark: World Health Organization Regional Office for Europé.

[nop2118-bib-0037] Yin, R. K. (2013). Case study research: Design and methods. Thousand Oaks, CA: Sage publications.

